# Reference values for urinary protein, albumin, beta 2-microglobulin, and the alpha 1-microglobulin-to-creatinine ratio in Japanese children

**DOI:** 10.1007/s10157-023-02392-4

**Published:** 2023-09-07

**Authors:** Shojiro Okamoto, Takeshi Matsuyama, Riku Hamada, Yoshihiko Morikawa, Masako Tomotsune, Tetsuji Kaneko, Katsumi Abe, Atsushi Uchiyama, Masataka Honda

**Affiliations:** 1 Department of Pediatrics, Tokai University School of Medicine, 143 Shimokasuya, Isehara, Kanagawa Japan; 2 Department of Pediatrics, Fussa Hospital, 1-6-1 Kamidaira, Fussa, Tokyo, Japan; 3 Department of Nephrology and Rheumatology, Tokyo Metropolitan Children’s Medical Center, 2-8-29 Musashidai, Fuchu, Tokyo, Japan; 4 Clinical Research Support Center, Tokyo Metropolitan Children’s Medical Center, 2-8-29 Musashidai, Fuchu, Tokyo, Japan; 5 Tokyo Health Service Association, 1-2 Ichigayasadohara, Shinjuku-Ku, Tokyo, Japan

**Keywords:** Children, Urinary screening, Reference values of urinary marker, Urinary protein, Urinary albumin, Urinary beta 2-microglobulin, Congenital anomalies of kidney and urinary tract

## Abstract

**Background:**

The importance of the ratio of creatinine to urinary protein, albumin, and low-molecular weight protein as a urinary marker in chronic kidney disease patients is widely recognized. However, no reference values have hitherto been established for these markers in Japanese children. The present study aimed to establish the reference values for these urinary markers in Japanese children.

**Methods:**

The first morning urine was randomly collected from 1712 pupils aged ≥ 3 to < 18 years during school and kindergarten mass urinary screenings. The upper limit of the reference values was set at the 97.5th percentile of the creatinine ratio per marker.

**Results:**

The urinary protein-to-creatinine ratio (PCR), urinary albumin-to-creatinine ratio (ACR), urinary beta 2-microglobulin-to-creatinine ratio (BMCR), and urinary alpha 1-microglobulin-to-creatinine ratio (AMCR) showed an age-related decrease at the 50th percentile reflecting an age-related increase in urinary creatinine. The appropriate reference value for the PCR and ACR was 0.12 g/gCr and 35 mg/gCr, respectively, in the entire cohort. The appropriate reference value for the BMCR was 0.5 μg /mgCr for age ≥ 3 to < 6 years and 0.35 μg/mgCr for age 6 years or older. The appropriate reference value for the AMCR was 5.0 μg/mgCr for age ≥ 3 to < 6 years and 3.5 μg /mgCr for age 6 years or older.

**Conclusion:**

The present study was the first to determine appropriate reference values for the PCR, ACR, BMCR, and AMCR based on an analysis of the first morning urine samples of a large number of children.

**Supplementary Information:**

The online version contains supplementary material available at 10.1007/s10157-023-02392-4.

## Introduction

The urinary protein-to-creatinine ratio (PCR) and urinary albumin-to-creatinine ratio (ACR) are essential for evaluating chronic kidney disease (CKD), and the importance of assessing urinary albumin in children is now attracting more attention. The 2012 Guidelines for CKD issued by Kidney Disease: Improving Global Outcomes (KDIGO) state that the urinary excretion of albumin is a risk factor of earlier deterioration of the glomerular filtration rate in pediatric patients as well as adults with CKD [1]. However, the same guidelines also indicate that there is no high-quality evidence for urinary albumin excretion in children, unlike for urinary protein excretion [1]. However, measuring low-molecular weight proteins in patients with congenital anomalies of the kidney and urinary tract (CAKUT) allows CKD progression to be evaluated [2–5]. Measuring the urinary excretion of low-molecular weight proteins is also recommended as means of detecting tubular diseases, such as Dent disease and nephronophthisis [1].

When evaluating CKD in children, the urinary Cr value and other markers need to be adjusted for age. However, as of yet no large-scale analysis of the PCR, ACR, urinary beta 2-microglobulin-to- creatinine ratio (BMCR) or urinary alpha 1-microglobulin-to-creatinine ratio (AMCR) has been conducted.

The present study aimed to establish age-appropriate reference values for the PCR, ACR, BMCR, and AMCR for the Japanese pediatric population.

## Materials and methods

### Materials

In Japan, the regional education boards and schools have had the responsibility of conducting annual school urinary screening for five decades. The screening is usually done in April at the start of each school year. Samples of the first morning urine are tested at testing centers designated by the regional education boards.

### Study sample

In the present study, an equal number of urine samples were collected from each age group and sex at screenings between April 8 and May 14, 2014. The subjects ranged in age ≥ 3 to < 18 years. The parameters for random selection were based on 333,411 urine samples analyzed by the Tokyo Health Service Association (THSA) in 2014. In total, 1712 samples, including samples from 866 male and 846 female subjects, were collected for the present study in cooperation with the THSA, which has been carrying out urinalysis for schools and kindergartens in wide areas of Tokyo prefecture. The Clinical Research Support Center at Tokyo Metropolitan Children’s Medical Center conducted the randomization and management of the samples. The data center informed the THSA about the urinalysis schedule, name of the screening venues, subjects’ age, sex, and expected sample number in advance and randomly selected the facilities, etc., to ensure that more than 50 samples were obtained per age group and sex.

### Measuring methods

Measurement of urinary creatinine (U-Cr), protein (U-Prot), albumin (U-Alb), beta 2-microglobulin (U-BMG), and alpha 1-microglobulin (U-AMG) was done within ten hours after collection. The urinary protein and albumin values were measured using the pyrogallol red method (Micro TP-AR, Eiken Chemical Co., Ltd., Tokyo) and immunonephelometry (LZ test U-ALB, Eiken Chemical Co., Ltd., Tokyo), respectively. Beta 2-microglobulin and alpha 1-micoroglobulin were measured using latex nephelometry (using LZ test β2-M and LZ test α1-M, Eiken Chemical Co., Ltd., Tokyo), and creatinine was measured using the enzymatic method. All measurements were performed by the Tokyo Health Service Association.

### Statistical analysis

The ratio of creatinine to urinary protein, albumin, beta 2-microglobulin, and alpha 1-mciroglobulin was calculated for each subject. The 97.5th percentile was established as the upper limit for the laboratory measurements in accordance with the recommendation of the Clinical and Laboratory Standards Institute [6]. The subjects were divided into the ≥ 3- to < 6-year-old, ≥ 6- to < 12-year-old, and ≥ 12- to < 18-year-old age groups corresponding to the pre-school age, pre-pubertal age, and adolescence, respectively. To establish clinically simplified reference values, percentile ranks were calculated for PCR, ACR, BMCR, and AMCR values per age group in considering values of approximated 97.5th percentiles. JMP15.2.1 “SAS Institute Japan” and EZR4.0.3 [7] were used for all statistical analyses. The Kruskal–Wallis test was used to compare the marker values among the age groups, and the Steel–Dwass method was used to adjust for multiplicity. The Mann–Whitney U test was used to assess differences between the sexes.

## Results

Table 1 shows the exact number of samples included in the present study. The samples were allocated evenly among the age groups and between the sexes. In total, 1712 samples comprising more than 50 and less than 78 samples per sex and age group were included for analysis.

**Table 1:** Number of subjects by age and sex

Age (year)	Male (number)	Female (number)	Total (number)
3	78	57	135
4	64	60	124
5	51	53	104
6	51	54	105
7	57	63	120
8	54	55	109
9	60	65	125
10	57	53	110
11	63	51	114
12	56	50	106
13	55	55	110
14	63	56	119
15	50	54	104
16	54	56	110
17	53	64	117
Total	866	846	1712

**Fig. 1:**
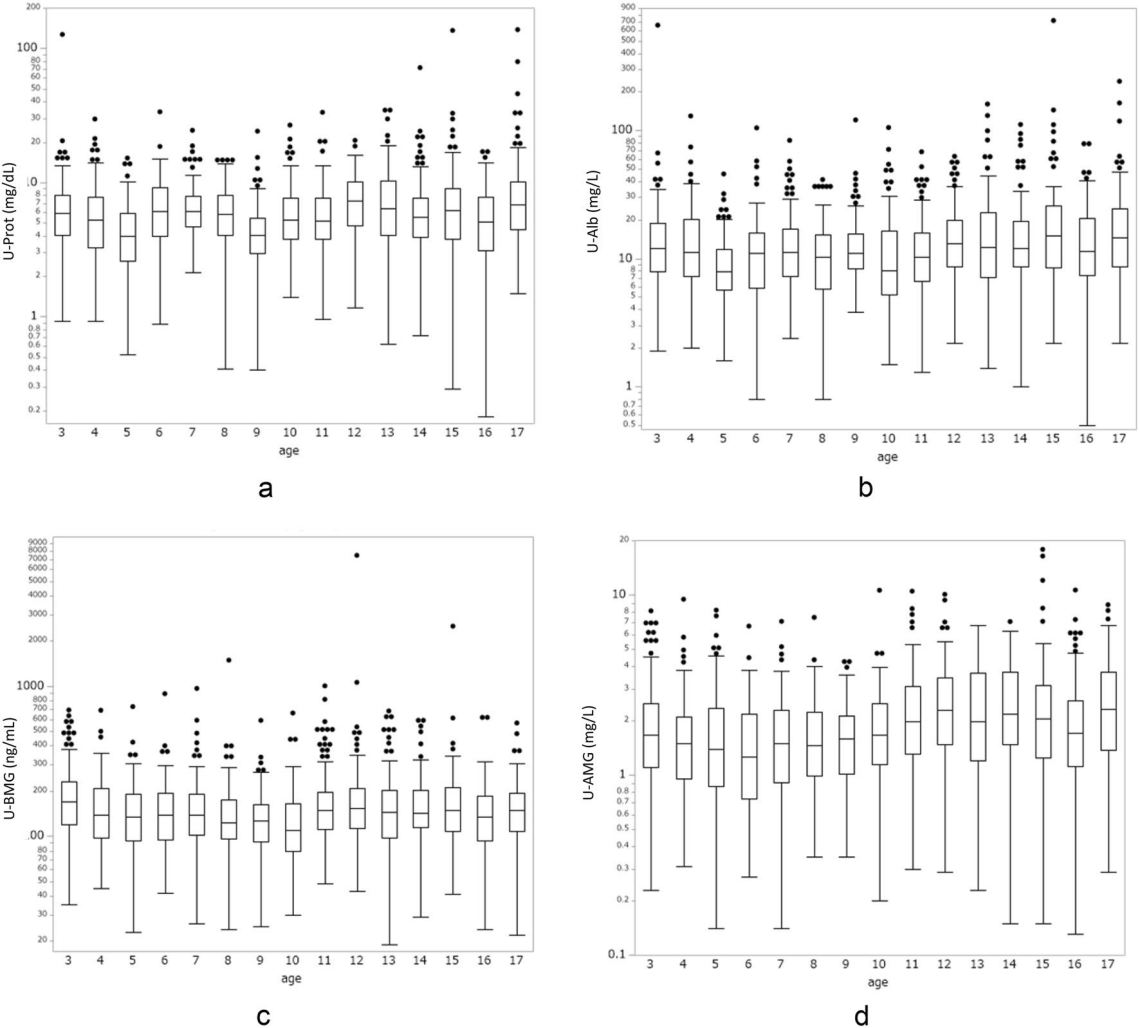
Box-and-whisker diagram with logged data by age. **a** U-Prot, **b** U-Alb, **c** U-BMG, **d** AMCR. The interquartile range is indicated in the boxes, and ± 1.5* the interquartile range is indicated within the lines. Values falling outside the quartiles are indicated by dots. U-Prot: urinary protein, U-Alb: urinary albumin, U-BMG: urinary beta 2-microglobulin, U-AMG: urinary alpha 1-microglublin

**Fig. 2:**
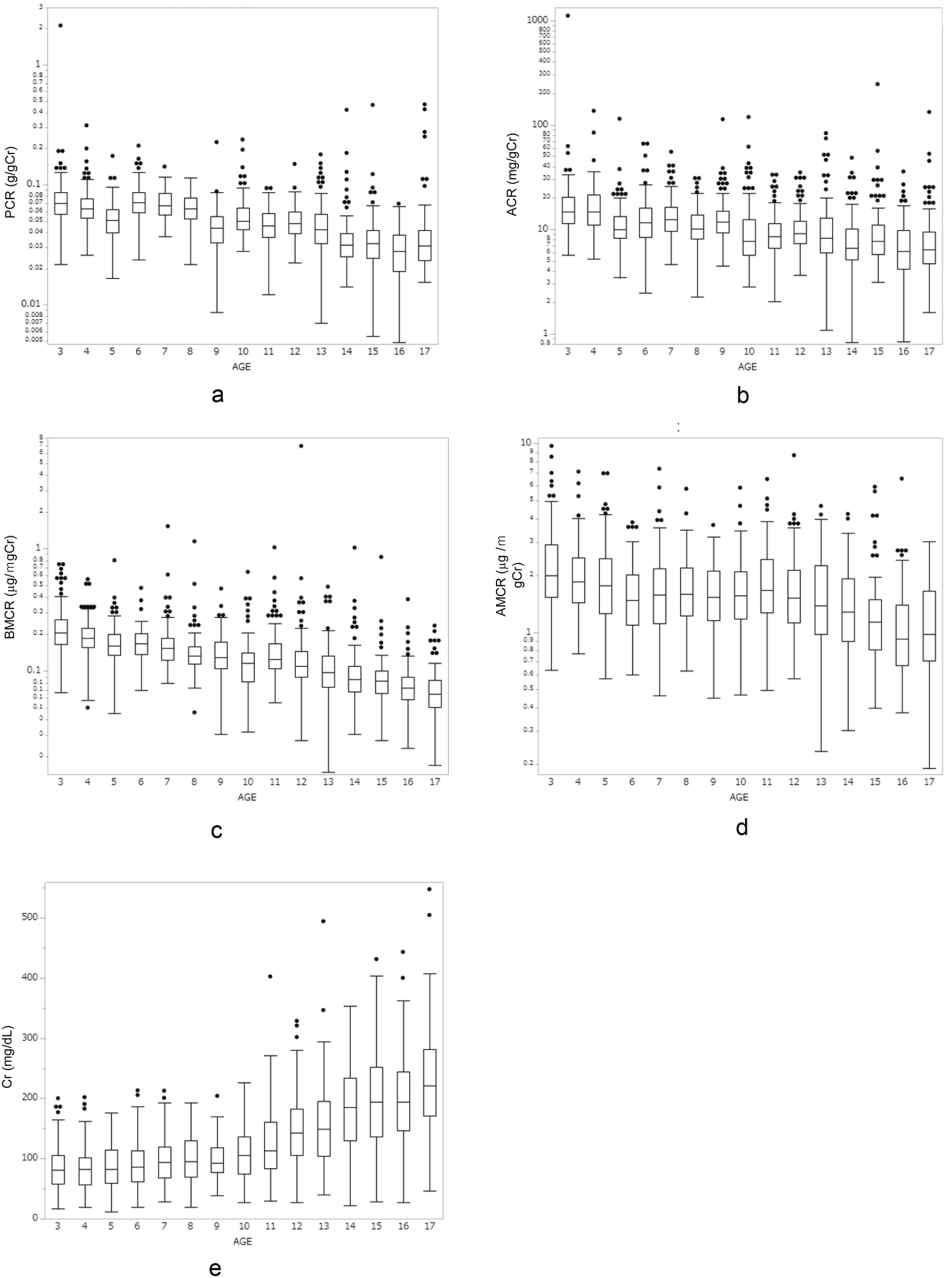
Box-and-whisker diagram with logged data by age. **a** PCR, **b** ACR, **c** BMCR, **d** AMCR, by age. **e** Box plot of U-Cr by age. The interquartile ranges are indicated in the boxes, and ± 1.5* the interquartile range is indicated within the lines. Outliers outside the quartiles are indicated by dots. PCR: urinary protein-to-creatinine ratio, ACR: urinary albumin-to-creatinine ratio, BMCR: urinary beta 2-microglobulin-to-creatinine ratio, AMCR: urinary alpha 1-microglobulin-to-creatinine ratio, U-Cr: urinary creatinine

Figure 1 shows a box-and-whisker diagram with logged data for U-Prot, U-Alb, U-BMG, and U-AMG, and Fig. 2 shows a box plot with logged data for the PCR, ACR, BMCR, AMCR, and U-Cr per age. Compared to the data in Fig. 1, the data in Fig. 2 show a general decrease in PCR, ACR, BMCR, and AMCR values with increasing age, which was reflected by increased urinary creatinine. Data on each marker and each marker divided by Cr were examined separately for each age group (Table 2 and Supplementary table). Table 2 shows the 50th, 90th, 95th, 97.5th, and 99th percentiles for the PCR, ACR, BMCR, and AMCR per age group. The Kruskal–Wallis test and Steel–Dwass test used to analyze the difference among these markers per age group demonstrated a significant decrease in all the markers with increasing age (*p* < 0.001). BMCR and AMCR clearly had a higher value for the 97.5th percentile in the ≥ 3- to < 6-year-old age group, unlike PCR and ACR.

**Table 2:** 50^th^, 90^th^, 95^th^, 97.5^th^, and 99^th^ percentile values for the Cr, PCR, ACR, BMCR, and AMCR in all the subjects per age group

	Percentile	Cr (mg/dL)	PCR (g/gCr)	ACR (mg/gCr)	BMCR (μg /mgCr)	AMCR (μg /mgCr)
All (*n* = 1712)	50th	112.6	0.05	9.8	0.12	1.51
	90th	237.2	0.09	21.4	0.23	2.85
	95th	277.4	0.10	27.8	0.29	3.54
	97.5th	309.2	**0.12**	**34.5**	**0.37**	**4.12**
	99th	343.1	0.17	53.0	0.55	5.55
≥ 3- to < 6-year-old (*n* = 363)	50th	81.5	0.06	13.3	0.18	1.85
	90th	130.0	0.10	26.3	0.30	3.68
	95th	154.0	0.11	30.4	0.35	4.34
	97.5th	167.7	**0.13**	**35.9**	**0.54**	**5.25**
	99th	185.7	0.19	71.0	0.62	7.03
≥ 6- to < 12-year-old (*n* = 683)	50th	97.8	0.06	10.6	0.14	1.58
	90th	161.1	0.09	20.8	0.23	2.74
	95th	181.7	0.10	27.5	0.28	3.25
	97.5th	195.7	**0.11**	**34.6**	**0.34**	**3.71**
	99th	213.6	0.14	44.0	0.48	4.68
≥ 12- to < 18-year-old (*n* = 666)	50th	182.1	0.03	7.5	0.08	1.22
	90th	288.5	0.06	17.0	0.16	2.49
	95th	320.9	0.08	22.8	0.21	2.96
	97.5th	342.7	**0.11**	**31.8**	**0.28**	**3.59**
	99th	404.0	0.18	51.6	0.38	4.21

Bold values indicate 97.5^th^ percentile value

*Cr* creatinine, *PCR* protein-to-creatinine ratio, *ACR* albumin-to-creatinine ratio, *BMCR* beta 2-microglobulin-to-creatinine ratio, *AMCR* alpha 1-microglobulin-to-creatinine ratio

Kruskal–Wallis test comparing PCR, ACR, BMCR, and AMCR in the three age groups. *p* < 0.001 for all items. The Steel–Dwass test was used to analyze the ≥ 3- to < 6-year-old group vs. the ≥ 6- to < 12-year-old group; the ≥ 3- to < 6-year-old group vs. the ≥ 12- to < 18-year-old group; and the ≥ 6- to < 12-year-old group vs. the ≥ 12- to < 18-year-old group. *p* < 0.001 for all the markers

Table 3 shows the results of the statistical analysis by sex. Male subjects in the ≥ 12- to < 18-year-old group tended to have a higher level of low-molecular weight proteins (BMCR and AMCR), and female subjects in the ≥ 6- to < 12- and ≥ 12- to < 18-year-old groups tended to have a higher ACR.

**Table 3:** Sex differences in Cr, PCR, ACR, BMCR, and AMCR in all the subjects per age group

Percentile		Cr(mg/dL)			PCR(g/gCr)			ACR(mg/gCr)			BMCR(μg/mgCr)			AMCR(μg /mgCr)		
		Male	Female		Male	Female		Male	Female		Male	Female		Male	Female	
All subjects	50th	110.1	115.9	*p* =0.41	0.05	0.05	*p* =0.499	9.3	10.4	*p* <0.001	0.13	0.12	*p* <0.001	1.64	1.4	*p* <0.001
	97.5th	321.1	297.9		0.12	0.12		30.5	37.7		0.39	0.32		4.48	3.82	
≥ 3- to < 6- year-olds	50th	81.7	80.6	*p* =0.806	0.06	0.06	*p* =0.237	13.3	13.4	*p* =0.918	0.18	0.18	*p* =0.739	1.94	1.82	*p* =0.952
	97.5th	172.2	163.7		0.12	0.14		33	38.2		0.55	0.5		5.01	6.2	
≥ 6- to < 12- year-olds	50th	96.8	98.7	*p* =0.76	0.054	0.059	*p* =0.0033	10.17	11.21	*p* <0.001	0.14	0.14	*p* =0.514	1.63	1.55	*p* =0.501
	97.5th	193.5	202.2		0.1	0.118		27.4	36.94		0.36	0.32		3.81	3.61	
≥ 12- to <18- year-olds	50th	181.3	183	*p* =0.903	0.034	0.035	*p* =0.882	6.33	8.8	*p* <0.001	0.089	0.079	*p* <0.001	1.54	0.93	*p* <0.001
	97.5^th^	347.9	329.8		0.136	0.084		31.21	35.95		0.38	0.177		4.18	2.61	

*Cr* creatinine, *PCR* protein-to-creatinine ratio, *ACR* albumin-to-creatinine ratio, *BMCR* beta 2-microglobulin-to-creatinine ratio, *AMCR* alpha 1-microglobulin-to-creatinine ratio

*p* values were analyzed using the Mann–Whitney *U* test

Percentile ranks were calculated for PCR, ACR, BMCR, and AMCR per age group to determine the simplified reference values (Table 4). For PCR and ACR, 0.12 g/gCr and 35.0 mg/gCr was approximated to the 97.5th percentile per age group. On the other hand, for BMCR, 0.35 μg/mgCr was approximated to the 97.5th percentile for the group aged 6 years or more while 0.5 μg /mgCr was closer to the 97.5th percentile for the group aged ≥ 3 to < 6-years. For AMCR, 3.5 μg /mgCr was closer to the 97.5th percentile for the group aged 6 years or more, while 5.0 μg /mgCr was closer to the 97.5th percentile for the group aged ≥ 3 to < 6 years. Each of these values was adopted as a reference value.

**Table 4:** Percentile rank at arbitrary designated values for PCR, ACR, BMCR, and AMCR

	≥ 3- to < 6-year-olds (*n* = 363)	≥ 6- to < 12-year-olds (*n* = 683)	≥ 12- to < 18-year-olds (*n* = 666)
PCR (g/gCr)^1)^			
Percentile rank at 0.10	0.910	0.948	0.969
Percentile rank at 0.12	0.960	0.982	0.980
Percentile rank at 0.15	0.982	0.992	0.987
ACR(mg/gCr) ^2)^			
Percentile rank at 30.0	0.943	0.962	0.965
Percentile rank at 35.0	0.971	0.975	0.980
Percentile rank at 40.0	0.981	0.988	0.983
BMCR (μg /mgCr) ^3)^			
Percentile rank at 0.30	0.902	0.962	0.976
Percentile rank at 0.35	0.948	0.976	0.980
Percentile rank at 0.50	0.968	0.99	0.994
Percentile rank at 0.60	0.989	0.993	0.995
AMCR (μg /mgCr) ^4)^			
Percentile rank at 3.0	0.808	0.933	0.951
Percentile rank at 3.5	0.874	0.963	0.973
Percentile rank at 4.0	0.932	0.984	0.984
Percentile rank at 5.0	0.967	0.992	0.994

*Cr* creatinine, *PCR* protein-to-creatinine ratio, *ACR* albumin-to-creatinine ratio, *BMCR* beta 2-microglobulin-to-creatinine ratio, *AMCR* alpha 1-microglobulin-to-creatinine ratio

Percentile rank at 0.10, 0.12, 0.15 g/gCr for PCR per age group, 2) Percentile rank at 30.0, 35.0, 40.0 mg/gCr for ACR per age group, 3) Percentile rank at 0.30, 0.35, 0.50, and 0.60 for BMCR μg/mgCr per age group, 4) Percentile rank at Cr 3.0, 3.5, 4.0, and 5.0 μg/mgCr for AMCR per age group

## Discussion

The present study established a reference value of 0.12 g/gCr for the PCR for the entire cohort; the Japanese Society of Nephrology’s adult reference value for PCR is 0.15 g/gCr. The appropriateness of the PCR reference value was particularly clear for those aged 6 years or older. The KDIGO guidelines and *Nelson Textbook of Pediatrics* recommend an ACR reference value of 30 mg/gCr [1, 8]; however, the findings of the present study suggest that 35 mg/gCr may be more appropriate for children older than 3 years. Until now, no reference values for the BMCR or AMCR have been published. The present study, which is the only large-scale study of these markers in the pediatric population to date, found that 0.5 μg /mgCr for the 3- to 5-year-old group and 0.35 μg /mgCr for children 6 years or older are adequate as reference values for the BMCR, while 5.0 μg /mgCr and 3.5 μg /mgCr are adequate as reference values for the AMCR for the respective groups. The result of dividing each value by the creatinine value is more important in clinical practice for reducing the effects of urine concentration. Moreover, the age difference in these markers becomes more apparent when their value is divided by the creatinine value, thereby yielding age-weighted values.

The Clinical and Laboratory Standards Institute recommends the 97.5th and the 2.5th percentiles as the upper and lower limits of the reference range, respectively [6]. The present study was able to establish the 97.5th percentile as an appropriate reference value for the PCR, ACR, BMCR, and AMCR (Table 2). Our data indicated that lower percentile figures were more appropriate for the BMCR and AMCR in the oldest age group; thus, specifying the reference value per age group may be necessary to avoid underdiagnosing kidney diseases even if thus far no studies have examined the need to adjust the reference values for age.

In previous guidelines and textbooks, the reference values for the PCR fell between 0.15 g/gCr and 0.2 g/gCr. The 2012 CKD guidelines published by KDIGO suggest that the PCR reference value should be < 0.2 g/gCr in the first morning urine in children older than 24 months [1]. The *Nelson Textbook of Pediatrics* also recommends 0.2 g/gCr Cr as the normal range for the PCR in the first morning urine in children older than 2 years [8] based on a study by Hogg et al. [9]. Our data suggested that modifications were necessary to establish reference values for the Japanese pediatric population, and in the present study the 97.5th percentile was adopted as an appropriate reference value most closely approximating in the percentile ranks. According to our data, the percentile rank at 0.15 g/gCr for the PCR fell between the 98th to 99th percentile per age group (Table 4), suggesting that the reference value of 0.15 g/gCr may lead to underdiagnosis of kidney diseases in older children. The reference value of 0.12 g/gCr may be more appropriate for all age groups, as shown in Table 4.

The conditions under which urine samples are collected affect the PCR value. The KDIGO guidelines and the *Nelson Textbook of Pediatrics* suggest collecting the first morning urine to rule out false positive results due to orthostatic proteinuria [1, 8]. Thus, in the present study, only the first morning urine collected at school screenings was used for analysis.

To date there are no studies of the ACR reference value for Japanese children. Based on the definition of albuminuria of the Nation Health and Nutrition Examination Survey III (NHANES III) [10], the KDIGO guidelines recommend an ACR reference value < 30 mg/gCr. The present study found the 97.5th percentile for ACR to fall between Cr 32 mg/gCr and 35 mg/gCr for all three age groups; based on this finding, 35 mg/gCr was chosen as an adequate ACR reference value (Table 4).

Low-molecular weight proteins can be markers of tubular injury or disorders and tend to occur at high levels in CAKUT, according to some previous studies [2–5, 11]. Assadi et al. found a correlation between the severity of vesicoureteral reflux and urinary BMG excretion [2]. Radhakrishna et al. found that 64% of patients with CAKUT had high urinary BMG excretion [5]. Although CAKUT is not generally considered a tubular disease, the primitive duct is characteristic of dysplastic kidneys and the involvement of the tubules and interstitium is widely recognized in several types of advanced CAKUT. These features may explain the increase in the excretion of low-molecular weight proteins in other CAKUT phenotypes. Although age-related adjustment of the reference values has yet to be done, a previous report by Hibi et al. [12] found evidence of changes related to age. In the present study, a decrease in the 50th percentile value due to the increasing excretion of U-Cr with age was observed for each marker (PCR, ACR, BMCR, and AMCR), and a clear, increasing tendency in the 97.5th percentile value of the BMCR and AMCR was observed in the youngest age group. Thus, it is appropriate to establish a discrete reference value for the AMCR and BMCR for these age groups. Table 4 shows the percentile rank for the PCR, ACR, BMCR, and AMCR in our study. Based on these data, we recommend 0.5 μg/mgCr and 0.35 μg /mgCr as a reference value for BMCR and 5.0 μg /mgCr and 3.5 μg/mgCr as a reference value for AMCR in the 3- to 5-year-old group and children 6 years or older, respectively.

Our study found differences by sex in the ACR, BMCR, and AMCR in the two, older age groups. A lower ACR in male subjects was reported by Muller et al. in 1999 [13], but this finding was not used to establish a range of appropriate reference values. The older male subjects in the present study had a higher AMCR, possibly as a result of higher serum α1-microglobulin [14]. However, no sex-related difference in serum or urine beta 2-microglobulin has been reported. CKD-related morbidities apparently differ by sex, but further study of CKD patients is needed to verify this finding.

The present study has several limitations. The urine samples were collected only in Tokyo; hence, there may be a sampling bias. However, the samples were taken from a wide swath of Tokyo, including rural areas. Hence, the differences in locality may not be meaningful. Children with kidney disease were not included in the present study. However, most of these children do not take part in mass school urinary screenings in Japan, and their exclusion therefore is unlikely to have affected the results of the present study.

## Conclusion

The present study established reference values for the PCR, ACR, BMCR, and AMCR in Japanese children by analyzing a large pediatric cohort. The present study is the first study of its kind to analyze the Japanese pediatric population and will be extremely useful for future clinical practice.

### Supplementary Information

Below is the link to the electronic supplementary material.

Supplementary file1 (DOCX 17 KB)
