# Correction to: Reference values for urinary protein, albumin, beta 2‑microglobulin, and the alpha 1‑microglobulin‑to‑creatinine ratio in Japanese children

**DOI:** 10.1007/s10157-023-02410-5

**Published:** 2023-10-21

**Authors:** Shojiro Okamoto, Takeshi Matsuyama, Riku Hamada, Yoshihiko Morikawa, Masako Tomotsune, Tetsuji Kaneko, Katsumi Abe, Atsushi Uchiyama, Masataka Honda

**Affiliations:** 1 Department of Pediatrics, Tokai University School of Medicine, 143 Shimokasuya, Isehara, Kanagawa Japan; 2 Department of Pediatrics, Fussa Hospital, 1-6-1 Kamidaira, Fussa, Tokyo, Japan; 3 Department of Nephrology and Rheumatology, Tokyo Metropolitan Children’s Medical Center, 2-8-29 Musashidai, Fuchu, Tokyo, Japan; 4 Clinical Research Support Center, Tokyo Metropolitan Children’s Medical Center, 2-8-29 Musashidai, Fuchu, Tokyo, Japan; 5 Tokyo Health Service Association, 1-2 Ichigayasadohara, Shinjuku-Ku, Tokyo, Japan

**Correction to: Clinical and Experimental Nephrology** 10.1007/s10157-023-02392-4

In the original publication, one of the abbreviations was incorrectly typed in the Results section of the Abstract.

The text “urinary beta 2-microglobulin-to-creatinine ratio (AMCR)” has been revised to read “urinary beta 2-microglobulin-to-creatinine ratio (BMCR)”.

Furthermore, there were some extraneous elements in Table 2 and Table 4.

In Table 2, the “e” at the bottom of the "Percentile" column has been removed.

In Table 4, the redundant age range descriptions (≥ 3- to < 6-year-olds; ≥ 6- to < 12-year-olds; ≥ 12- to < 18-year-olds) have been deleted, and the alignment has been corrected.

The original article has been corrected.

