# Supplementary material for: Reference values for urinary protein, albumin, beta 2-microglobulin, and the alpha 1-microglobulin-to-creatinine ratio in Japanese children
Source: Clin Exp Nephrol. 2023 Sep 7;28(1):50–7. doi: 10.1007/s10157-023-02392-4 (PMC10766671; doi:10.1007/s10157-023-02392-4)
Supplement: Supplementary file 1 — Supplementary file1 (DOCX 17 KB) [file 10157_2023_2392_MOESM1_ESM.docx]

Reference values for urinary protein, albumin, beta 2 microglobulin, and the alpha 1 microglobulin-to-creatinine ratio in Japanese children

Shojiro Okamoto^1)^, Takeshi Matsuyama^2)^, Riku Hamada^3),^ Yoshihiko Morikawa^4)^, Masako Tomotsune^4)^, Tetsuji Kaneko^4)^, Katsumi Abe^5)^, Atsushi Uchiyama^1)^, Masataka Honda^4)^

Corresponding Author

Name: Masataka Honda, MD

E-mail address: mhond@fol.hi-ho.ne.jp

Telephone: (+81) 42-300-5111

Fax: (+81) 42-312-8162

Supple. Table 50th and 97.5th percentile values for the U-Cr, U-Prot, U-Alb, U-BMG and U-AMG per age group

|  | | U-Cr (mg/dL) | U-Prot (mg/dL) | U-Alb(mg/L) | U-BMG (ng/mL) | U-AMG (mg/L) |
| --- | --- | --- | --- | --- | --- | --- |
| ≥ 3 to < 6-year-olds | 50th percentile | 81.45 | 5.06 | 10.20 | 145 | 1.51 |
|  | 97.5th percentile | 167.67 | 15.12 | 40.05 | 441 | 5.75 |
| ≥ 6 to < 12-year-olds | 50th percentile | 97.76 | 5.33 | 10.55 | 133 | 1.55 |
|  | 97.5th percentile | 195.75 | 15.11 | 41.89 | 419 | 4.64 |
| ≥ 12 to <18-year-olds | 50th percentile | 182.09 | 6.21 | 13.00 | 146 | 2.08 |
|  | 97.5th percentile | 342.74 | 22.10 | 77.28 | 499 | 6.67 |

U-Cr: urinary creatinine, U-Prot: urinary protein, U-Alb: urinary albumin, U-BMG: urinary beta 2-microglobulin, U-AMG: urinary alpha 1-microglobulin.
